# The African American Wellness Village in Portland, Ore

**Published:** 2006-06-15

**Authors:** Corliss McKeever, Nancy Koroloff, Collaine Faddis

**Affiliations:** African American Health Coalition, Inc; Regional Research Institute, Graduate School of Social Work, Portland State University, Portland, Ore; African American Health Coalition, Inc, Portland, Ore

## Abstract

More than 80% of African Americans in Oregon reside in the Portland metropolitan area; African Americans comprise 1.7% of the state's population. Although relatively small, the African American population in the state experiences substantial health disparities. The African American Health Coalition, Inc was developed to implement initiatives that would reduce these disparities and to promote increased communication and trust between the African American community and local institutions and organizations. One of these initiatives is an annual Wellness Week featuring an African American Wellness Village. The Wellness Village uses a model of cultural sensitivity to provide access to free health screenings, links between health care organizations and African American community members, and health education and information.

The African American Health Coalition, Inc obtained a Racial and Ethnic Approaches to Community Health (REACH) 2010 grant to sustain this programming. The Wellness Village is supported by five major sponsors; annual attendance has ranged from 700 to 900 participants. The African American Health Coalition's evaluation of the event indicates that more than 50% of respondents identify the Wellness Village as the only place that they receive health screenings. Participants with access to screenings elsewhere report that a culturally sensitive environment that inspires trust is the reason they prefer the screenings offered at the Wellness Village.

Culturally sensitive health fairs such as the Wellness Village may play an important role in bringing preventive health screenings to African American communities. Collaboration between black and white health care providers is critical in this effort. Partnerships must be built at multiple levels, including institutions to provide financial resources and in-kind donations, community members to assist with outreach and recruitment, and health care professionals to conduct screenings and services.

## Health Disparities Among African Americans in Oregon

The African American community in Portland, Ore, is clearly defined and is centralized in the Portland Metropolitan Statistical Area (MSA), which is located in the northwestern part of the state. The MSA encompasses the city of Portland, the largest city in the state, and surrounding suburbs in Multnomah, Washington, and Clackamas counties. More than 40% of the state's population and 80% of African Americans living in Oregon reside in the Portland MSA. Of the four million Oregonians in 2005, 68,000 were African American (1).

In Oregon, 20.2% of African Americans do not have health insurance, whereas only approximately 14% of the state population is uninsured (2). The Oregon Health Plan (OHP), Oregon's Medicaid program, provides coverage to individuals and families below the federal poverty level. In 2005, African Americans represented only 1.7% of the state's total population, but they accounted for 4.6% of participants receiving OHP benefits (3). 

A disparity also exists in cardiovascular health. Although the picture of cardiovascular disease (CVD) for African Americans is bleak nationwide, in Oregon the data are even more striking. For example, from 1996 to 2000, African Americans in Oregon had a higher stroke death rate than the national stroke death rate for African Americans (209 per 100,000 compared with 166 per 100,000) (4,5). African Americans are also less likely than their white counterparts to have ever been told that their blood cholesterol or blood pressure is high and that it is treatable (6).

Although the national health disparities of African Americans were documented by the landmark *Report of the Secretary's Task Force on Black and Minority Health* (7), many people believed that such disparities were unlikely in Oregon because of the small African American population. Because of Oregon's small African American population, the Behavioral Risk Factor Surveillance System data from the 1990s did not identify a health disparity. However, the Oregon Health Division did a statewide analysis in the early 1990s comparing birth and death rates of blacks and whites, and the report's findings indicated substantial disparities (8).

The African American Health Coalition, Inc (AAHC) was founded by volunteers in 1989 to call attention to such health disparities, develop initiatives to reduce them, and increase the trust of Portland's African American community in local health authorities and health care systems. One of AAHC's initiatives is the African American Wellness Village, held annually as part of African American Wellness Week. Introduced in 1996, the Wellness Village is a health fair that offers health screenings and health promotion information for African Americans in a culturally sensitive environment. The Wellness Village provides a trusted source of information for participants and the opportunity to communicate with health care providers in a comfortable setting.

## The African American Experience in Portland

The African American experience in Portland is rooted in displacement and intergenerational mistrust of local authorities and health care systems. This mistrust affects the way in which the African American community views its hospitals, doctors, health care systems, and local authorities. Oregon's original constitution contained an exclusion law prohibiting African Americans from permanent residency. In the 1860s, African Americans who migrated to Oregon faced widespread prejudice and intolerance and were forced to live in a small area of land along the Columbia River (9).

During World War II, an influx of African Americans arrived to work in the ship yards (9). This migration resulted in a severe housing shortage exacerbated by the city's refusal to grant building permits to African Americans. To provide housing for their workers, the Kaiser Shipbuilding Company erected Vanport, a housing development situated on land reclaimed from the Columbia River and surrounded by dikes (9,10). In 1948, local officials evacuated Vanport because of a threat of flooding. After receiving an all-clear from local authorities, African Americans returned to their homes, only to have them be flooded by the Columbia River. Vanport was completely destroyed, and 25% of the 18,000 people left homeless were African American (11). The Vanport flood increased many African Americans' mistrust of local authorities; that mistrust still exists. Following the flood, many African Americans moved to a small area of prime property in the neighboring Portland community of North/North East Albina. This neighborhood was developed to contain thriving, vibrant, black-owned businesses, churches, social clubs, and civic organizations.

In 1970, a major health care system approached Albina community homeowners, offering to purchase their property at below market value. Most community members had no experience with land development, so when approached with a cash offer, they sold their homes. Homeowners were also promised that a large health center would be built on the land, which would enhance the community's quality of life. This promise was not fulfilled, and many African American families and businesses were once again displaced.

Since 1989, state and local health departments, researchers, and community groups have studied African American health conditions in Oregon and the African American community’s perceptions of its health care services (12). There are several themes that emerge from data gathered from focus groups, stories, and responses to participant questionnaires. These themes include the following: 1) the need for education (e.g., “We don’t know,” “We were never told,” “We didn’t understand”); 2) the effect of stress (e.g., “We don’t feel comfortable,” “We can’t talk about it”); and the impact of racism (e.g., “We are not treated the same,” “We don’t have the same access,” “Nothing is designed to meet our needs”). The AAHC developed the Wellness Village to meet these needs and concerns.

## The African American Wellness Village Model

### Program structure

The AAHC uses a model of cultural sensitivity as described by Resnicow et al (13) for the Wellness Village. A culturally sensitive program is defined by both its surface structure and deep structure. The surface structure matches a program to identifiable characteristics of the target population (e.g., food, people, language, music) to increase its acceptance of the program and its messages. Deep structure incorporates the cultural, social, historical, and environmental determinants of health behavior of the target population into the program and influences outcomes of a health program. The Wellness Village program and its target population are matched for communication styles, historical experiences, and culture.

### Health screenings

Health screenings are provided for free at the Wellness Village by 15 to 20 of the AAHC's collaborative partners. The screenings provided by our health care partners include the following: dental, by the Oregon Chapter of Lion's Club International; HIV and hepatitis C, by the Multnomah County Health Department; blood pressure, cholesterol, and diabetes, by the American Heart Association; and glaucoma, by Devers Eye Institute. The Oregon Immunization Program offers reduced-cost flu shots paid for by the AAHC for high-risk groups, including seniors and infants. Each health care partner is required to commit staff to be available for questions from community members the entire day of the event.

### Activities and programs

The Wellness Village also offers cultural and educational programming. The opening ceremony includes a calling of the elders, libations, and African drumming. Child care is provided in the Children's Hut by volunteers and staff. The Wellness Village also provides a healthy soul-food cooking demonstration, and attendees receive food samples. Attendees learn how to add spices to soul food to enhance flavor but reduce the fat. Participants can also take salsa and African dance lessons, go for the afternoon Wellness Walk through the neighborhood, or enjoy the gospel choir. Door prizes are given away at the end of the event. Throughout the day, attendees can stroll among the health screening and education booths.

Participants also receive information about year-round AAHC health promotion interventions supported by the Centers for Disease Control and Prevention's (CDC's) Racial and Ethnic Approaches to Community Health (REACH)Participants also receive information about year-round AAHC health promotion interventions supported by the Centers for Disease Control and Prevention's (CDC's) Racial and Ethnic Approaches to Community Health (REACH) 2010 initiative. The REACH 2010 initiative consists of four interventions: 1) Lookin' Tight Livin' Right, a beauty and barber shop intervention based on peer education that uses motivational interviewing and relationships among hair stylists to educate their clients about CVD; 2) Healthier Options to Living Longer Actively (HOLLA), a train-the-trainer model that uses students to deliver health education messages; 3) Wellness Within REACH, a partnership with parks and recreational facilities that provides access to free physical activity classes; and 4) Spice It Up, a partnership with the Oregon Food Bank to provide ethnic cooking classes to African American community members. The REACH 2010 program provides year-round access to health education that follows up on information that participants receive at the Wellness Village.

## Development of the Wellness Village

In the early 1990s, the AAHC called attention to the Oregon Health Division's analysis, which found large health disparities for African Americans in Oregon. The AAHC alerted local media, and newspapers published data showing the disproportionately high infant mortality rates of African Americans. The newspaper coverage gained the attention of state decision makers and resulted in offers to collaborate. Partnerships with Legacy Health Systems, Kaiser Permanente, Providence Health System, Oregon Health & Science University, Eli Lilly and Co, and the state and county health departments provided funding and technical assistance for the first African American Wellness Village in 1996. Since then, the event has been held annually, and the original health care systems continue to provide financial support.

The overwhelming community response to the first Wellness Village was the catalyst that led the coalition volunteers to see the need to build an infrastructure and become a nonprofit corporation. The first step of institutional capacity building was realized by the formation of the 501(c)(3) in December 1998. The second step was to open an office and secure four grants from state and local health departments to address issues of HIV, tobacco use, breast cancer, cervical cancer, and diabetes. Subsequently, a multidisciplinary team developed a proposal to CDC's REACH 2010 program. A community action plan was developed to address CVD, the leading cause of death among African Americans in Oregon, and its risk factors, as well as diabetes and smoking. A grant was awarded to the AAHC in September 2000.  

### Partnerships

The Wellness Village is supported statewide. A proclamation in 1996 declared the third week in October "African American Wellness Week." The African American Wellness Village attracts partners who do not typically have connections to the African American community. The Wellness Village was initially funded with in-kind donations from coalition members and a major sponsorship from Kaiser Permanente. Since its early years, the Wellness Village has had five major sponsors who annually contribute $10,000 each: Legacy Health System, Oregon Health & Science University, Providence Health System, Kaiser Permanente, and Eli Lilly and Co. 

The Wellness Village was founded by a group of volunteers that included black health professionals and white advocates who united to fight the social injustice that is a factor in health disparities. The initial success of the Wellness Village was based on the community and the health care system's trust in the black health care professionals and their white colleagues, who opened doors and provided access to resources. These partnerships developed primarily through personal and business relationships forged by black health care professionals. The black health care professionals maintained respect and connections with local African Americans, while at the same time gaining recognition and credibility within the health care community, including from the original major sponsors and state and local health departments. 

In addition to the major sponsors, other partners contribute substantially to the village by offering donated resources for raffles, free screenings, and education. Exhibitors purchase tables for $200 each; they secure a table by filling out an application at least 3 months before the event. Each organization is required to define the resources and services to be offered. The AAHC carefully screens each application to avoid exploitation of the community and the participants. Exhibitors agree to report required data on the screenings they perform.

### Timeline

The planning process for the Wellness Village begins in the first quarter of the year with the recruitment of an annual Wellness Village planning team. The planning team consists of representatives from each major sponsor; the AAHC management and Wellness Village coordinator; community members, including youths; staff members from local and state health care departments; and volunteer screeners. During this period, new sponsorships are identified and solicited, and program and recruitment materials are completed. During the second quarter, the planning team develops letters, flyers, and programs that will inform community members about the event. In the third quarter, the planning team finalizes the Wellness Village layout and event logistics and orders all of the needed supplies and equipment, including tables, props, T-shirts, and programs. All in-kind donations for raffles and prizes are solicited, and volunteers are recruited and trained. The fourth quarter is dedicated to community outreach through local contacts and the dissemination of flyers. The event timeline is the responsibility of the AAHC program coordinator, who records minutes, makes reminder calls, follows up with the planning team, and oversees the logistics.

### Recruitment

The Portland African American community is easily mobilized when approached by individuals whom they trust. Our methods for announcing the upcoming Wellness Village and recruiting participants include providing information to 22 exercise venues, using the AAHC Board and staff's social support networks, making presentations, and soliciting other social service agencies who also recruit participants through their constituencies. The African American community in Portland is tight-knit and is like a rural area in that word of mouth is a communications method often used (14). A media campaign in the local African American newspapers is launched 3 weeks before the event.   

## Consequences

Average attendance at the Wellness Village each year ranges from 700 to 900 community members. For the past 3 years, the AAHC has subcontracted with research evaluators who use surveys and observation to examine the impact of the Wellness Village. Between 2002 and 2004, 483 Wellness Village participants voluntarily completed a survey as they exited the village. Analyses of 4 years of data provide a rich view of the trends in participant needs and concerns. 

During the survey period, 66% to 78% of individuals completing surveys reported that the information received at the Wellness Village was "very helpful." Participants reported that they found information about nutrition, exercise, and healthy cooking most useful. Cooking demonstrations were added in response to these survey findings. Thirty percent to 50% of respondents also wanted or needed information about diabetes.

Each year, most attendees receive free screenings for one or more health conditions. The most frequently used screenings include blood pressure, vision, and glaucoma. More than 50% of the survey respondents said that the Wellness Village was the only place they received screenings. Qualitative responses suggest that participants come to the Wellness Village for screenings because they trust the AAHC and because the services are free. When health problems are identified, participants are linked to community heath care resources by the screeners. Participants are encouraged to visit the onsite OHP screeners to determine whether they qualify for Medicaid support. For participants who do not qualify for OHP and cannot pay for services, the Wellness Village and AAHC education and support groups are crucial.

The social aspects of the Wellness Village are very important, and many respondents commented on the community feeling of the event and on the friendliness of vendors and volunteers. Because so many people mentioned the social aspect of the Wellness Village, emphasis has been put on events such as church choir performances, African dancing, salsa dance lessons, and food tasting. Over the years, AAHC has responded to the community's feedback and added requested screenings, demonstrations, and exhibitors. The most effective mechanism for disseminating information about the Wellness Village has been word of mouth, with almost 40% of the respondents reporting that they had heard about the event from family or friends or at church.

Although the Wellness Village is a very important event, it is only part of the overall AAHC effort to eliminate health disparities. People who come to the Wellness Village often learn about or are already involved in another AAHC program. Approximately one third of respondents to a survey in 2004 reported that they were involved in the free exercise program, and approximately one third reported that they had participated in the Wellness Walk.

## Interpretation

By conducting an annual event that African Americans can rely upon, the AAHC has sustained a culturally specific event that delivers health education, preventive screenings, and links to resources and referral information. The Wellness Village provides a connection to services for community members who otherwise would not learn about or receive those services. Although the screeners are not exclusively African American, they are introduced to the community by the AAHC as trusted gatekeepers. For some individuals, the Wellness Village is the only venue where they can obtain annual health screenings. It continues to be a major community event.

One challenge of an annual event is to keep the volunteers, vendors, and staff engaged over time. The AAHC strategy includes personal contact and ongoing communication through newsletters, telephone calls, and involving our partners in other activities of the coalition. Direct feedback from vendors, volunteers, staff, and participants is used in the planning process for the next year. Because of the annual planning process, such an event requires a full-time staff to adhere to the time line.

Because of budget cuts to the OHP, more participants are screened each year for whom there are no community services. The AAHC has no mechanism for tracking people who are screened at the Wellness Village to determine whether they have been linked to needed services. Continued development of community partnerships may alleviate this problem. Despite criticism, health fairs may play an important role in bringing preventive health services to the African American community (15). Confrontational relationships can evolve into respectful collaborations over time. Collaboration between black and white health care providers at a personal level is a critical building block for such an event. Partnerships must be built on multiple levels, including institutions with financial resources and in-kind donations, community members to assist with outreach and recruitment, and professional health care volunteers to conduct screenings and services.
